# Developing aptamer probes for acute myelogenous leukemia detection and surface protein biomarker discovery

**DOI:** 10.1186/1756-8722-7-5

**Published:** 2014-01-09

**Authors:** Mingli Yang, Guohua Jiang, Wenjing Li, Kai Qiu, Min Zhang, Christopher M Carter, Samer Z Al-Quran, Ying Li

**Affiliations:** 1 UF/Shands Medical Laboratory at Rocky Point, 4800 35th Drive, Gainesville, FL 32608, USA

**Keywords:** Acute myeloid leukemia, Aptamer, Biomarker, Cell-SELEX, Siglec-5

## Abstract

**Background:**

The majority of patients with acute myelogenous leukemia (AML) still die of their disease. In order to improve survival rates in AML patients, new strategies are necessary to discover biomarkers for the detection and targeted therapy of AML. One of the advantages of the aptamer-based technology is the unique cell-based selection process, which allows us to efficiently select for cell-specific aptamers without knowing which target molecules are present on the cell surface.

**Methods:**

The NB4 AML cell line was used as the target cell population for selecting single stranded DNA aptamers. After determining the affinity of selected aptamers to leukocytes, the aptamers were used to phenotype human bone marrow leukocytes and AML cells in clinical specimens. Then a biotin-labelled aptamer was used to enrich and identify its target surface protein.

**Results:**

Three new aptamers were characterized from the selected aptamer pools (JH6, JH19, and K19). All of them can selectively recognize myeloid cells with Kd in the low nanomole range (2.77 to 12.37 nM). The target of the biotin-labelled K19 aptamer probe was identified as Siglec-5, a surface membrane protein in low abundance whose expression can serve as a biomarker of granulocytic maturation and be used to phenotype AML. More importantly, Siglec-5 expression can be used to detect low concentrations of AML cells in human bone marrow specimens, and functions as a potential target for leukemic therapy.

**Conclusions:**

We have demonstrated a pipeline approach for developing single stranded DNA aptamer probes, phenotyping AML cells in clinical specimens, and then identifying the aptamer-recognized target protein. The developed aptamer probes and identified Siglec-5 protein may potentially be used for leukemic cell detection and therapy in our future clinical practice.

## Introduction

Acute myelogenous leukemia (AML) is a heterogeneous group of malignant hematopoietic neoplasms derived from hematopoietic stem cells postulated to arise due to mutations of genes that regulated the orderly proliferation, differentiation, and maturation of hematopoietic cells. In the past two decades, scientific advances utilizing molecular techniques and cytogenetic detection have yielded new insights into the genetic and biologic features of acute leukemia. Despite these advances, the majority of patients who suffered from AML still died of their disease [1-3]. With the exception of a subtype of AML, AML M3 (i.e. acute promyelocytic leukemia, APL), we have not yet succeeded in translating our scientific discoveries into more effective treatments for the majority of AML patients. While therapeutic intensification, improved supportive care, and bone marrow transplantation have led to gradual improvements of outcome in children and younger adults with AML, the overall survival rate approaches 50%. In older individuals (>55-60 years) and in secondary AML patients, the outlook is more dismal with overall survival rates of 10-15% [4], typically attributed to an increase in unfavorable cytogenetic features.

Currently, immunophenotyping via immunohistochemistry and flow cytometric analysis plays a pivotal role in the detection and diagnosis of AML. However, the surface biomarkers currently used for immunophenotyping AML are adapted from the advancement of immunology research in the last several decades instead of being specifically developed for leukemic cell detection. While we use these biomarkers in our daily practice to classify leukemia into myeloid or lymphoid lineages, they neither identify the molecular events underlying the neoplastic processes nor provide adequate insight into the aggressiveness or prognosis of these diseases. As a consequence, many different leukemic variants become grouped together under the same name due to the lack of adequate biomarkers for effective stratification, despite not representing the same exact disease by nature or behavior [5,6]. In addition, when only a small number of leukemic cells are present it is often difficult, if not impossible, to determine the disease status due to their immunophenotypic similarity to normal cells. This is often the case following chemotherapy when minimal residual leukemia is present. Therefore, a new strategy using molecular aptamers is envisioned to discover biomarkers and apply them in clinical practice to improve therapeutic efficiency and the survival rate of AML patients.

Molecular aptamers consist of single stranded DNA or RNA that can recognize target proteins, peptides, and other small molecules. Through a process called SELEX (Systematic Evolution of Ligands by Exponential enrichment) [7-11], DNA or RNA aptamers specific for a known protein of interest can be selected from a random pool of oligonucleotide sequences, and then used as diagnostic and therapeutic reagents [12]. Traditionally, the targets (most often proteins), in most instances, have to be identified first before specific molecular probes, including monoclonal antibodies, can be developed. However, using live cells from leukemic cell lines, we established a unique cell-based selection process (Cell-SELEX) that allows for the selection of aptamers that can recognize live leukemic cells from patients [13]. Most importantly, the Cell-SELEX method allows us to select a group of cell-specific aptamers in a relatively short time period and selected aptamers can readily be tested and verified in clinical specimens, without knowing which target molecules are present on the cell surface. Thus far, while many DNA or RNA aptamers have been selected against various types of cells, a few surface proteins targeted by individual aptamers of interest were identified [14], which is probably due to the technical challenge in purification and identification of low-abundance membrane proteins [14]. The few reported proteins identified through individual aptamer probes include pigpen from the rat endothelial cell line YPEN-1 [15], Tenascin-C of U251 glioblastoma cells [16], immunoglobulin heavy mu chain in Burkitt’s lymphoma cells [17] and protein tyrosine kinase-7 (PTK7) on CCRF-CEM T-cell acute lymphoblastic leukemic cells [18].

In order to develop biomarkers for AML, we intended to design a pipeline approach for biomarker discovery: 1) To employ the Cell-SELEX technique to select for DNA aptamer probes against live leukemic cells; 2) To test selected aptamers by phenotyping normal human bone marrow cells or leukemic cells in clinical specimens; 3) To identify target proteins on leukemic cell surfaces with meaningful molecular signatures as demonstrated with the aptamers. In this study, we selected aptamers against NB4 AML cells. More importantly, with biotin-labelled aptamers we were able to demonstrate that the target protein for one of the new aptamers was a member of the sialic-acid-binding immunoglobulin-like lectins (Siglecs). Then the aptamer recognizing Siglec-5 was used to detect small numbers of AML cells in human bone marrow specimens.

## Material and methods

### Cell culture and Reagents

NB4 and HL60 human leukemic cell lines were obtained from ATCC (American Type Culture Collection, Manassas, Virginia) and were cultured in RPMI 1640 medium (Thermo Scientific HyClone, South Logan, Utah) supplemented with 10% fetal bovine serum (FBS) (heat inactivated, Thermo Scientific HyClone, South Logan, Utah), and antibiotics (100 units/ml penicillin-Streptomycin from Fisher BioReagents, Fairlawn, NJ). Before binding to aptamers, cells were washed with phosphate-buffered saline (PBS). The buffer used for aptamer binding and selection was prepared by adding 4.5 g/L glucose, 5 mM MgCl2, 0.1 mg/ml yeast tRNA (Fisher BioReagents, Fairlawn, NJ, USA) and 1 mg/ml Bovine Serum Albumin (BSA) (Fisher BioReagents) into Phosphate buffered saline (PBS) [13]. The used fluorochromes include allophycocyanin (APC), fluorescein isothiocyanate (FITC), phycoerythrin (PE), and peridinin chlorophyll protein (PerCP).

### Cell-SELEX procedures for aptamer selection

HPLC purified libraries (Sigma-Aldrich, St. Louis, MO) contain a segment of randomized sequence of 50 nucleotides (nt) flanked by PCR primer hybridization sites (5′-GACGCTTACTCAGGTGTGACTCG-50nt-CGAAGGACGCAGATGAAGTCTC-3′). Biotinylated PCR-primers were used in the PCR reactions for the synthesis of biotin-labelled DNA molecules. After heat denaturation at 95°C for 5 min, the denatured DNAs were placed on ice immediately and the biotinylated strands were separated from the complement strands by streptavidin-coated magnetic beads (Thermo Scientific Pierce, Pittsburgh, PA).

The selection processes were performed similarly as described before [13,19]. 20 nmoles of synthesized single stranded DNA pool were dissolved in 1 ml of binding buffer and used for the first round selection. 100–200 pmoles of pool dissolved in 400 μL binding buffer were used for the remaining rounds of selection. The DNA pools were denatured by heating at 95°C for 5 min and placed on ice for 10 min before binding. The single stranded DNA pool was incubated with 1–2 × 10^6^ target (NB4) cells on ice for 1 hr. After washing, the bound DNAs were eluted by heating at 95°C for 5 min in 400 μL of 2 mM Tris–HCl buffer (pH 8.0). The eluted DNAs were amplified by PCR (Taq-polymerase and dNTP’s were obtained from Fisher (Fairlawn, NJ)) for 25–30 cycles of 0.5 min at 94°C, 0.5 min at 60°C, and 0.5 min at 72°C, followed by 5 min at 72°C. The amplified sense DNA pool used for the next round selection was separated from antisense PCR products by streptavidin-coated magnetic beads. After 8–20 rounds of selection, biotinylated selected DNA pools or single stranded DNA control were bound to NB4 cells on ice for 30 min, washed twice with cold PBS and then incubated with 5 μL PE-streptavidin (0.5 mg/ml, Becton Dickinson, NJ) on ice for 30 min. After washed twice with PBS, the NB4 cells were subject to flow cytometry analysis. After the selected pool showed significantly higher fluorescence signals than the unselected one (see Additional file 1: Figure S1), selected pool was PCR-amplified using unlabeled primers, cloned into pPCR-Script Amp SK(+) vector with PCR-Script Amp Cloning Kit (Agilent Technologies, San Diego, CA) and transformed into Escherichia coli (DH5α), as described in previous studies [13,19]. 100 white colonies were picked and grew for minipreprations of plasmid DNA with QIAprep Spin Miniprep Kit (Hilden, Germany). The DNA sequences were determined by the DNA sequencing facility at the Interdisciplinary Center for Biotechnology Research, University of Florida. DNA sequences that were present in more than two clones were considered as aptamer candidates.

### Flow cytometric analysis of aptamer binding to target cells

Biotin-labelled, selected single stranded DNA pools or individual aptamers of interest were incubated with 5 × 10^5^ cells in 200 μL of binding buffer with 0.1% NaN3 on ice for 30 min. Cells were washed twice with 4 ml of PBS buffer and incubated with 5 μL PE-streptavidin (0.5 mg/ml, Becton Dickinson, NJ) for 30 min. Biotin-labelled unselected library was used as a negative control. The cells were washed once and cell-bound fluorescence was determined with a FACScan or FACSCalibur flow cytometer (Becton Dickinson, NJ) by counting 20,000-50,000 events. The FITC, PE and PERCP were activated by blue laser (488 nm) and APC by red laser (635 nm). Fluorescence-labelled monoclonal antibodies were used with aptamers to define lineages of bone marrow leukocytes and leukemic cells in clinical specimens. To determine the binding affinity of selected aptamers, all experiments for the aptamer binding assay were repeated 2–4 times. The GraphPad Software (San Diego, CA, USA) was used to analyze the data for obtaining the equilibrium dissociation constants (Kd) of the fluorescent aptamers and the 95% confidence interval.

### Clinical sample preparation and testing

All clinical samples were submitted for pathological evaluation to the Shands Hospital Hematopathology Laboratory, University of Florida. The studies were approved by the University of Florida Institutional Review Board. The presented data include thirty-six cases of AML. Ten cases of non-malignant human bone marrow were also used for the studies.

Erythrocytes in all bone marrow samples specimens were lysed as described before [20]. Human bone marrow or leukemic cells were immunophenotyped with thirty fluorochrome-conjugated monoclonal antibodies in our clinical flow cytometry panels [20], for immunophenotyping mature or immature granulocytes, monocytes, blasts and lymphocytes so that we can determine how to selectively gate the cell population of interest. The data analysis was performed using FCS Express software (De Novo Software, Los Angeles, CA, USA, http://www.denovosoftware.com). Initial cell subpopulations were established using the levels of CD45 expression and side-scatter (SSC) properties [21,22]. After defining immunophenotypes of leukemic cells, antibodies for CD45, CD34, CD117, CD33, HLA-DR, CD64 or CD14 (Becton Dickinson, NJ) were used to select cells of interest to determine fluorescence levels of bound aptamers for individually gated subpopulations.

### Statistical analyses

GraphPad Software was used for statistical analyses. The One-way Analysis of Variance (ANOVA) or *T* test was used to compare fluorescence levels of aptamers bound on the different cell populations. Unless stated otherwise, results were given as mean ± standard deviation (SD) and the P values were also given for comparison as necessary.

### Protease treatment for cells

NB4 cells (5 × 10^6^) were washed with PBS and then incubated with 1 ml of 0.25% trypsin/0.1% EDTA in Hank’s buffered salt solution (HBSS) (Thermo Scientific HyClone, Pittsburgh, PA) at 37°C for 10 min. FBS was then added to quench the protease. After washing with PBS, the treated cells were used for aptamer-binding assays as described earlier.

### Enrichment and identification of the aptamer-bound target protein

A total of, 8 × 10^8^ NB4 cells in the active growing phase were harvested, and used as target cells for aptamer K19 binding followed by enrichment of the aptamer-bound target protein. The NB4 cells were pre-incubated with 8 ml of RPMI media containing 1 mg of heat-denatured Herring Sperm DNA (Promega) at 4°C for 15 min to block potential nonspecific binding of the aptamer to the cells. The cells were then incubated in the binding buffer with or without biotin-labelled aptamer K19 (at the final concentration of 300 nM) and the binding was performed without any aptamers was used as a negative control. To determine the specificity of aptamer binding, an additional negative control was made by pre-incubating the cells with 300 nM of the unlabeled K19 aptamer for 1 hr prior to the binding of the biotin-labelled aptamer. After binding, the cells were washed three times with PBS to remove the unbound aptamer. A small aliquot of each cell sample (5 × 10^5^ cells) was taken, and analysed by flow cytometry with PE-streptavidin to monitor the aptamer binding.

The aptamer-bound or control cells were then lysed in 10 ml of lysis buffer containing 10 mM HEPES pH 7.4, 150 mM NaCl, 1% Triton X-100 and 1 mM EDTA plus HaltTM protease inhibitor cocktail (Thermo Scientific Pierce, Pittsburgh, PA) on ice for 15 min. After centrifugation at 14000 g for 15 min, the supernatant was incubated with 1 mg (100 μl) of magnetic streptavidin beads at 4°C for 30 min to capture the protein-aptamer complexes. The beads with bound aptamer-protein complexes were then collected on an EasySep magnet stand (Stemcell Technologies, Vancouver, BC, Canada) and washed five times with 15 ml of the lysis buffer. The enriched proteins were heated for elution and separated by 11% SDS-polyacrylamide gel electrophoresis (SDS-PAGE). The gels were then silver-stained with the Pierce Silver Stain Kit (Thermo Scientific Pierce, Rockford, IL). The aptamer-specific protein bands were excised and trypsin-digested in situ [23] and analysed by QSTAR LC-MS/MS and a MASCOT database search at the Interdisciplinary Center for Biotechnology Research Mass Spectrometry Core Facility, University of Florida.

### Studies of aptamer-antibody competition

Fluorescein-conjugated mouse monoclonal anti-human Siglec-5 (Clone 194128, R&D Systems, Minneapolis, MN, USA) and biotin-labelled or unlabeled K19 aptamers were used in the competition studies. Competition experiments were carried out in two ways: 1) NB4 cells (2 × 10^5^) were incubated with 300 nM of the unlabeled K19 aptamer or a control aptamer in 100 μL of binding buffer at 4°C for 45 min. After washing with PBS to remove the unbound aptamers, cells were incubated with 5 μg/ml fluorescein-conjugated anti-Siglec-5 antibody or control IgG1 antibody in 50 μL of PBS with 0.5% BSA at 4°C for 45 min. After washing off of the unbound antibodies, the cells were analysed by flow cytometry. 2) The NB4 cells were incubated with the anti-Siglec-5 or the control antibody and then with the biotin-labelled aptamer K19 or control aptamers. After PBS washing, PE-streptavidin was added followed by flow cytometric analysis as described earlier.

### Non-Radioactive Cell Proliferation Assay

CellTiter 96® Non-Radioactive Cell Proliferation Assay Kit (Promega, Wisconsin) was used to determine viable cell numbers after NB4 cells were incubated with various amounts of aptamer-streptavidin-saporin complexes or mixtures of aptamer and unlabeled saporin. After incubation for 72 hours, the assay is performed by adding a premixed, optimized Dye Solution to culture wells of a 96-well plate. In 4-hour incubation, living cells convert the tetrazolium component of the Dye Solution into a formazan product. The Solubilization/Stop Solution then was added to the culture wells to solubilise the formazan product, and the absorbance at 570 nm is recorded using a 96-well plate reader.

## Results

### Using Cell-SELEX for selection of aptamers bound to NB4 cells

**Figure 1:**
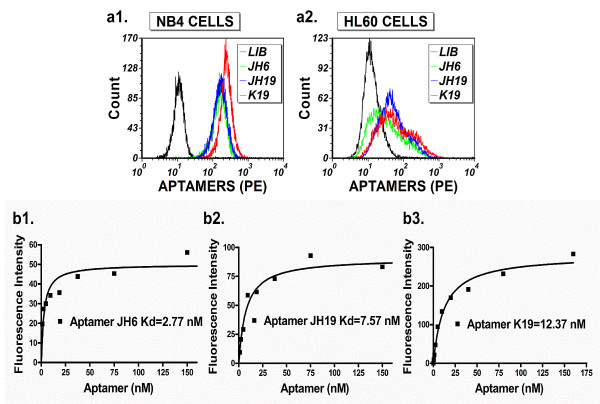
**Aptamer recognition of cultured NB4 and HL60 leukemic cells. (a)**. Comparison of aptamer recognition of cultured NB4 and HL60 leukemic cells. Individually synthesized biotin-labelled aptamers and PE-streptavidin were analyzed with flow cytometry in order to compare their ability to recognize NB4 and HL60 cells. Single-stranded library DNA was used as a negative control. The binding of selected aptamers with cells is illustrated as the following: negative control (black); JH6 (green); JH19 (blue); K19 (red). The final concentration of these aptamers in binding buffer was 150 nM. **(b)**. Determination of the aptamer binding affinities to NB4 cells. The biotin-labeled aptamers and PE-labeled streptavidin were used for the binding assays. The background binding was measured by using unselected single-stranded library DNA. The fluorescence intensity geometric means of bound aptamers was determined by flow cytometry. The equilibrium dissociation constants (Kd) of the fluorescent ligands were obtained by fitting the dependence of specific binding fluorescence intensity on the concentration of the ligands to the Equation Y = Bmax*X/(Kd + X) using the GraphPad Software (San Diego, CA, USA) as described in previous studies [13].

Cultured AML NB4 and HL60 cell lines have been used for aptamer selection, and aptamers selected against HL60 cells can recognize monocytic cells [19]. Because of previous unsuccessful attempts to select aptamers against NB4 cells, we focused on the viability of the cultured cells used for aptamer selection. Through careful optimization, we critically improved the cell culture conditions necessary to maintain NB4 cells in the active proliferation phase. The use of cells in the active proliferation phase improved the cells viability during the aptamer selection procedures, which in turn reduced nonspecific aptamer binding caused by dead cell fragments or debris. As a result, we were able to select a panel of aptamers for NB4 cells. In addition, we significantly reduced the time period needed for Cell-SELEX, and were able to obtain the aptamers with approximately eight rounds of selection. 10 aptamer candidates were obtained through sequencing 100 individual clones and we chose three representative aptamers (JH6, JH19 and K19) (Table 1) for further studies because the three new aptamers showed much better recognition to NB4 cells than to HL60 cells, and the bound aptamers exhibited up to 8 to 22 fold increases in fluorescence intensity compared to the DNA library control (Figure 1a). We then determined the affinity of the three aptamers to NB4 cells. All the three aptamers have high affinity for NB4 cells with calculated Kd of 2.77 nM for JH6, 7.57 nM for JH19 and 12.37 nM for K19 (Figure 1b and Table 1).

**Table 1:** Sequences and binding affinity of selected aptamers

**Aptamers**	**Kd (nM)**	**Confidence interval (nM) (95%)**	Aptamer sequences*
**JH6**	2.77	0.69-4.85	5′-GTACGCCGCAAGACGAGTTGTGTATAAGCCGGC-3′
**JH19**	7.57	4.13-11.02	5′-AGGTGTGACTCGATCTGTGGGGGTT GGGGGGTGGTTTTTCGGAA-3′
**K19**	12.37	7.79-16.94	5′-AAGGGGTT GGGTGGGTTT ATACAAATTA ATTAATATTGTATGGTATATTT-3′

*All aptamers have two short sequences (5′GACGCTTACTCAGGTGTGACTCG3′ and 5′CGAAGGACGCAGATGAAGTCTC3′) attached to the 5′ and 3′ ends, respectively, of the aptamer sequences shown above. The short sequences at 5′ and 3′ ends were used for PCR amplification.

### The selected aptamers can differentially recognize myeloid cells in normal human bone marrow specimens

**Figure 2:**
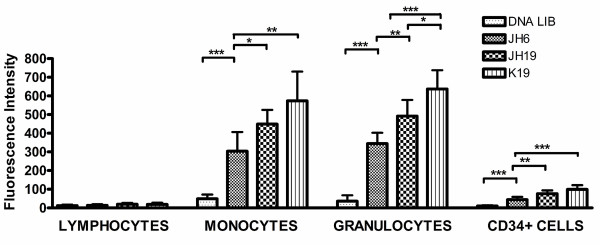
**Recognition of normal bone marrow leukocytes by aptamers JH6, JH19, and K19.** The fluorescence intensity of bound aptamers or single-stranded negative control DNA on normal human bone marrow cells, including lymphocytes, granulocytes, monocytes, and CD34+ cells was determined by flow cytometry. Fluorescence intensity is shown as mean ± standard deviation, and “*”, “**”, and “***” represent the *P* values of < 0.05, < 0.01, and < 0.001, respectively.

Because all three aptamers were selected against the AML NB4 cell line, we tested whether the selected aptamers (JH6, JH19 and K19) have an ability to recognize different types of leukocytes in human bone marrow specimens. While no binding on lymphocytes was seen, all the three aptamers showed high levels of binding (6 to 17 folds of fluorescence intensity over background) on mature and immature granulocytes and monocytes (Figure 2). The results suggest that the three aptamers may recognize myeloid-specific surface markers. The bound aptamer K19 had higher fluorescence intensity on granulocytes, monocytes, and NB4 cells than bound aptamers JH6 and JH19 (Figures 1 and 2). In addition, all three aptamers had low, but statistically significant, levels of binding on CD34(+) early hematopoietic precursors (Figure 2).

### The selected aptamers can differentially recognize leukemic cells from AML non-M3 and AML M3 cases

**Figure 3:**
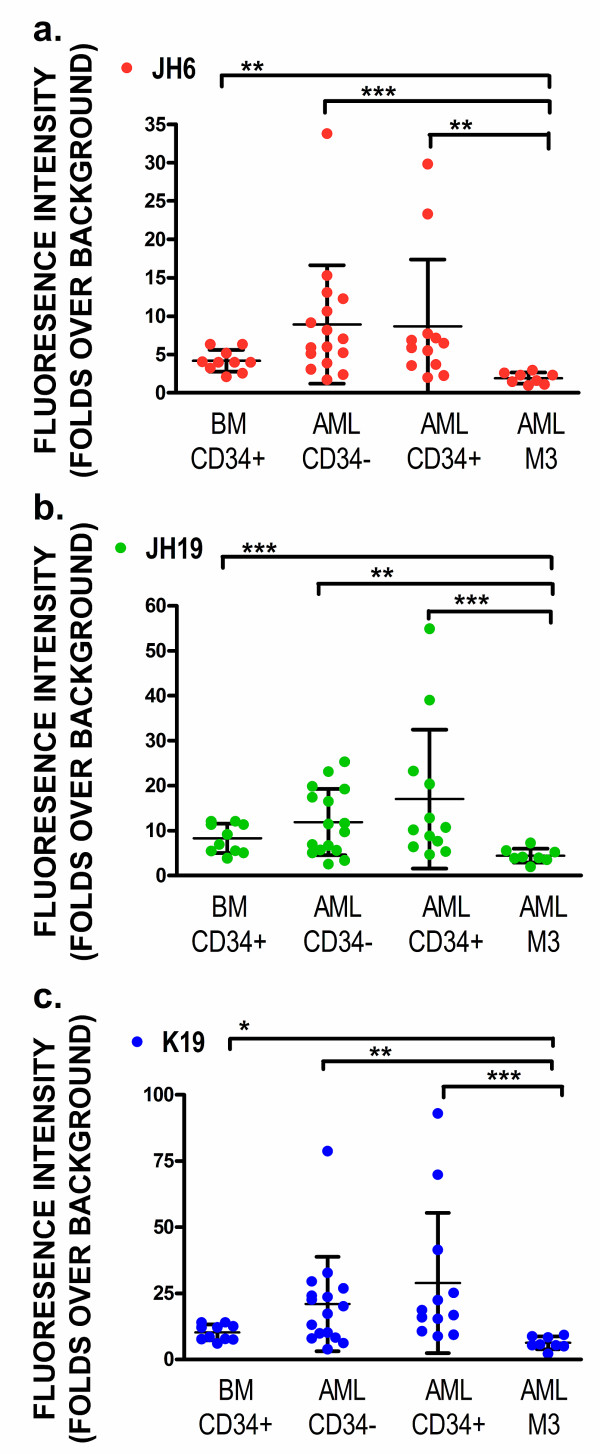
**Comparison of aptamer recognition of AML leukemic cells and non-malignant CD34(+) cells.** The AML cases were separated into three groups: 1) CD34(+) AML non-M3; 2) CD34(-) AML non-M3; and 3) AML M3. The fluorescence levels of bound aptamers or single-stranded negative control DNA were determined by flow cytometry. The fluorescence intensity levels of bound aptamers (folds over background) were calculated (**a**, JH6; **b**, JH19 and **c**, K19). Individual values for each aptamer bound on each case are shown as individual symbols, and mean ± standard deviation of individual groups are also shown. The *P* values are given as “*”, “**”, and “***” representing the *P* values of < 0.05, < 0.01, and < 0.001, respectively.

Because the three aptamers recognized maturing granulocytes and monocytes better than CD34(+) early progenitors, we separated AML clinical specimens into three groups: 1) AML non-M3 CD34(+); 2) AML non-M3 CD34(-); and 3) AML M3. We then determined if aptamers JH6, JH19, and K19 could differentially recognize any groups of AML cases. While these aptamers showed low levels of reactivity on normal CD34(+) progenitors, all three aptamers can recognize both CD34(+) and CD34(-) cells of AML non-M3 cases with the median values of fluorescence intensity being ~8 to 30 fold higher than those of background binding (Figure 3). However, the levels of the three aptamers bound on AML non-M3 cases varied significantly, and there was no statistical significance in aptamer binding levels between the normal CD34(+) cells and leukemic cells from AML non-M3 cases. Critically, all three aptamers had much lower levels of binding on leukemic cells of AML M3 cases than normal CD34(+) early progenitors or leukemic cells of AML non-M3 cases. These differences were statistically significant (Figure 3).

### Using biotin-labelled K19 aptamers to enrich and identify its target protein

**Figure 4:**
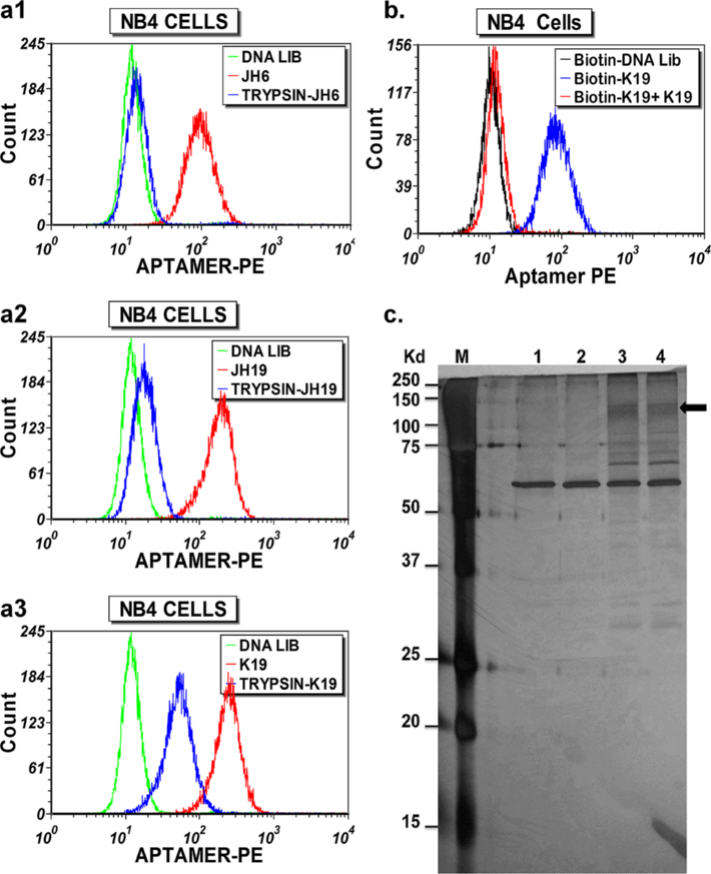
**Using biotin-labeled K19 aptamer to enrich its target protein. (a)**. Effect of trypsin pre-treatment on binding of aptamers to NB4 cells. NB4 cells were treated with trypsin at 37°C for 10 min. The binding of selected aptamers with trypsin-treated NB4 cells is illustrated: JH6 (a1); JH19 (a2); and K19 (a3). The final concentration of these aptamers in binding buffer was 300 nM. The background binding was measured by using biotinylated single-stranded negative control DNA. **(b)**. Binding competition of biotinylated-K19 to NB4 cells by the unlabeled K19 aptamers. NB4 cells were pre-incubated with the unlabeled K19 aptamers (300 nM) for 1 hr prior to binding of the biotin-labeled aptamers. The fluorescence intensity of bound aptamers is shown by the histograms (blue, binding of biotinylated K19; red, binding of biotinylated K19 after blocking by non-labeled K19). The background binding was measured by using biotinylated single-stranded negative control DNA. **(c)**. Silver-stained polyacrylamide gel electrophoresis (SDS-PAGE) separating the proteins captured by aptamer K19. NB4 cells were pre-incubated with or without the unlabeled aptamer K19 for 1 hr prior to binding of the biotin-labeled aptamers. The NB4 cells were then lysed. The protein-aptamer DNA complex(s) were captured by the magnetic streptavidin beads, and were then separated by SDS-PAGE followed by silver staining for detection of characteristic protein bands. Lane M, molecular markers; Lane 1, proteins captured with streptavidin beads (no aptamer); Lane 2, proteins captured by biotin-aptamer K19 after blocking by unlabeled aptamer K19; Lanes 3 and 4, protein captured with biotinylated aptamer K19.

In order to determine if the targets of the aptamers may represent surface proteins or moieties associated with surface membrane proteins, we treated NB4 cells with trypsin before binding the aptamers on cells. As shown in Figure 4a, the binding sites of aptamers JH6, JH19 and K19, as indicated by the fluorescence intensity of bound aptamers, were partially or almost completely abolished by 10 min of trypsin digestion. These results suggest that the target molecules recognized by these aptamers may be directly or indirectly related to surface proteins anchored on the cell membrane.

Since aptamer K19 bound NB4 cells demonstrate relatively higher fluorescent intensity, suggesting more abundant aptamer K19 binding sites as compared to the cells bound with aptamers JH6 and JH19, and three aptamers showed similar binding patterns when applied to bone marrow CD34(+) cells, granulocytes and monocytes, we focused on identification of the protein target associated with the binding site of aptamer K19. Flow cytometric analysis is a very sensitive technology, and we estimated that there were only a few hundred aptamer K19 binding sites on individual NB4 cells when we compared the fluorescence intensity of K19 to those of PE-beads (QuantiBRITE PE, Becton Dickinson), which are designed to estimate the number of bound antibody molecules per cell.

To verify the specific binding of aptamer K19 during target protein enrichment, we used a negative control, in which unlabeled aptamer K19 was used to block the binding of biotin-labelled aptamer K19 to NB4 cells. Flow cytometric analysis of small aliquots of the aptamer-bound cell samples, which were made to enrich target proteins, demonstrated that the unlabeled aptamer can completely abolish the binding of biotinylated ones, indicative of the binding specificity of aptamer K19 (Figure 4b).

The protein-aptamer complexes were extracted with the buffer containing 1% Triton X-100, captured using streptavidin-coated magnetic beads, and separated by SDS-PAGE. We then applied silver-stain for protein detection (Figure 4c). Compared with the negative controls (lane 1, without aptamer added and lane 2, blocked by unlabeled aptamer K19), several apparent K19-specific protein bands were shown in lanes 3 and 4. These bands were excised for further trypsin treatment, and analysed by mass spectrometry (MS). It is noteworthy that SDS-PAGE analyses were run under both reducing and non-reducing conditions, and the smear band at 130–140 kDa obtained under the non-reducing condition was reproducibly detected (Figure 4c, arrow).

**Figure 5:**
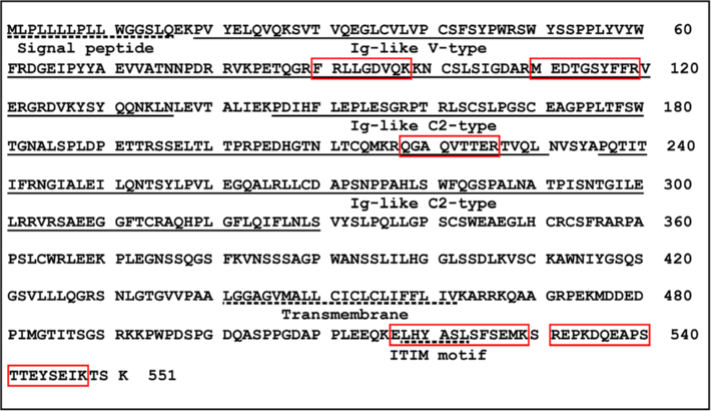
**The sequences of Siglec-5 protein and peptides recovered by mass spectrometric analysis of aptamer K19-enriched proteins.** The Ig-like V-type domain and two Ig-like C2-type domains of Siglec-5 are underlined while the signal peptide, the transmembrane region, and the immunoreceptor tyrosine-based inhibitory (ITIM) motif are marked with dotted underlines. The peptides identified by mass spectrometry are highlighted by bordering.

The MS data of peptides were used to search the MASCOT database in order to identify possible protein candidates. The candidate protein hits (~20) include many RNA or DNA binding proteins, intracellularly localized soluble proteins such as lysozyme C, and contaminated keratins. The only cell surface protein identified on the list was Sialic acid-binding Ig-like lectin 5 (Siglec-5). The MS data showed 5 unique peptides identified as fragments of Siglec-5. The sequences of identified peptides are marked on the Siglec-5 sequence as shown in Figure 5. Siglec-5 exists as a disulfide-linked dimer of ~140 kDa [24,25], which is in agreement with the size of the K19-bound 130–140 kDa protein band identified on SDS-PAGE under the non-reducing condition (Figure 4c, arrow).

### Aptamer K19 and anti-Siglec-5 antibody can compete against each other for the binding sites on the NB4 cells

**Figure 6:**
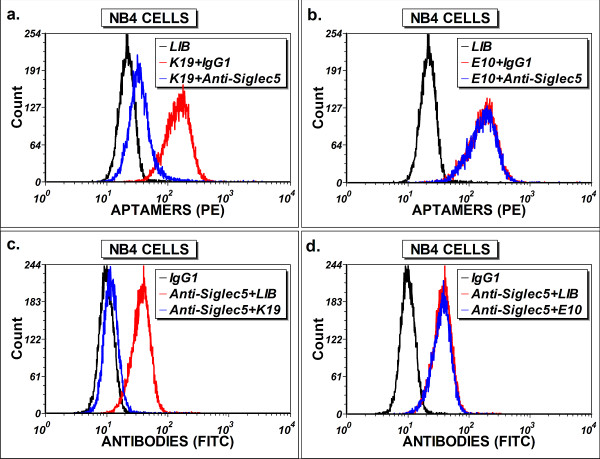
**Competition binding assays of aptamer K19 and anti-Siglec-5 antibody.** For blocking aptamer binding with antibodies, NB4 cells were pre-incubated with anti-Siglec-5 or isotype control antibodies before binding to biotin-labelled aptamers K19 **(a)** or E10 **(b)**. The fluorescence intensities of bound aptamers were determined by flow cytometry. Similar approaches were employed with aptamers K19 **(c)** or E10 **(d)** used to block binding of the FITC-anti-Siglec-5 antibody on NB4 cells. The FITC-isotype control (IgG1) was used as a negative control for specific antibody binding.

To confirm that Siglec-5 is the protein target of the aptamer K19, we carried out the competition experiment using a fluorescein-conjugated anti-human Siglec-5 antibody. As shown in Figure 6a and c, the aptamer K19 and the Siglec-5 antibody can compete against each other for the binding sites on the NB4 cells. In contrast, the control aptamer E10, which can also bind to NB4 cells (unpublished results), does not display any competition with the Siglec-5 antibody (Figure 6b and d), and the reactivity of aptamer K19 toward NB4 cells was not affected by isotype control antibodies (Figure 6a). Thus, we confirmed that Siglec-5 is the targeted protein recognized by aptamer K19, and that the binding site of aptamer K19 on the Siglec-5 protein may be sterically close to the epitope bound by the Siglec-5 antibody.

### Siglec-5 can be used as a biomarker for granulocytic maturation and AML cell detection as well as be used as a potential target for leukemic cell growth inhibition

**Figure 7:**
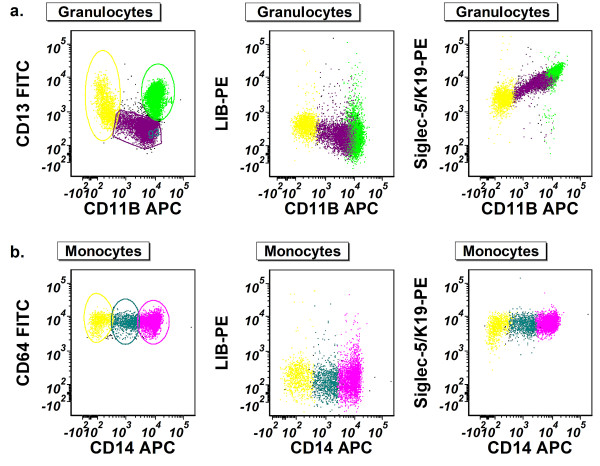
**Using Siglec-5 as a biomarker for granulocytic different maturation in human bone marrow.** Leukocytes from normal bone marrow aspirates were incubated with aptamer K19 or the single-stranded negative control DNA. **(a)**. Siglec-5 expression of maturing granulocytes. The granulocyte population was first identified using levels of side-scattered light (SSC) in combination with the fluorescence intensity of CD45. Then, maturing granulocytes were separated into three subsets according to the expression levels of CD13 and CD11b (left panel): early stage (yellow), immediate stage (purple), and matured stage (green). Fluorescence intensity (PE) of the granulocyte subsets bound with single stranded DNA control (middle panel) or aptamer K19/Siglec-5 (right panel) is shown in relation to fluorescence levels of CD11b (APC). **(b)**. Siglec-5 expression of maturing monocytes. The mature and immature monocytes were first identified using levels of side-scattered light (SSC) in combination with the fluorescence intensity of CD64. Then, maturing monocytes were separated into three subsets according to the expression levels of CD64 and CD14 (left panel): early stage (yellow), immediate stage (teal) and matured stage (fuchsia). Fluorescence intensity (PE) of the monocyte subsets bound with single-stranded negative control DNA (middle panel) or aptamer K19/Siglec-5 (right panel) is shown in relation to fluorescence levels of CD14 (APC).

Siglec-5 was reported to be expressed on granulocytes [26], but its expression during granulocytic or monocytic maturation has not been well characterized. Since aptamer K19 recognized maturing granulocytes much better than CD34(+) early progenitors in normal human bone marrow (Figure 2), we further determined whether its binding sites (i.e. Siglec-5 protein levels) on granulocytes vary during granulocytic maturation. By flow cytometric analysis, we separated maturing granulocytes or monocytes into three subsets: early stage, immediate stage, and matured stage, according to the expression levels of CD13 and CD11b for granulocytes and CD64 and CD14 for monocytes (Figure 7, left panel) [27-29]. We then determined the fluorescence levels of aptamer K19 bound on each subset. Compared with the negative control, the fluorescence intensity of bound aptamer K19 on granulocytes gradually increased during granulocytic maturation (Figure 7, right panel), indicating progressive up-regulation of Siglec-5 levels during granulocytic maturation. However, persistently high levels of Siglec-5 expression were observed on both CD64(+)/CD14(-) immature and CD64(+)/CD14(+) mature monocytes.

**Figure 8:**
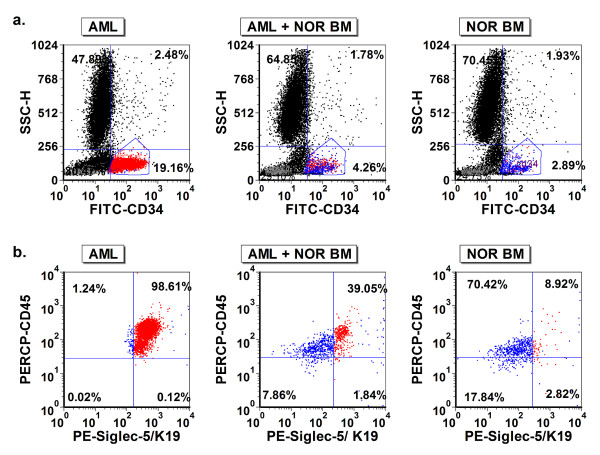
**Using Siglec-5 as a biomarker to detect low levels of AML cells in human bone marrow specimen.** A representative AML bone marrow (left panels in **a**. and **b**.) and normal bone marrow (NOR BM) (right panels in **a**. and **b**.) case are shown. In addition, the AML specimen is diluted with normal bone marrow cells (middle panels in **a**. and **b**.), and the final AML cell fraction was approximately 1.6%. CD34(+) cells in three samples are gated selectively according to the levels of CD34 and SSC **(a.)**, and the expression of Siglec-5 on CD34(+) cells is displayed in **(b.)** The CD34(+) leukemic cells (red dots in **b**.) have higher levels of Siglec-5 than normal CD34(+) cells in normal bone marrow (blue dots in **b**).

**Figure 9:**
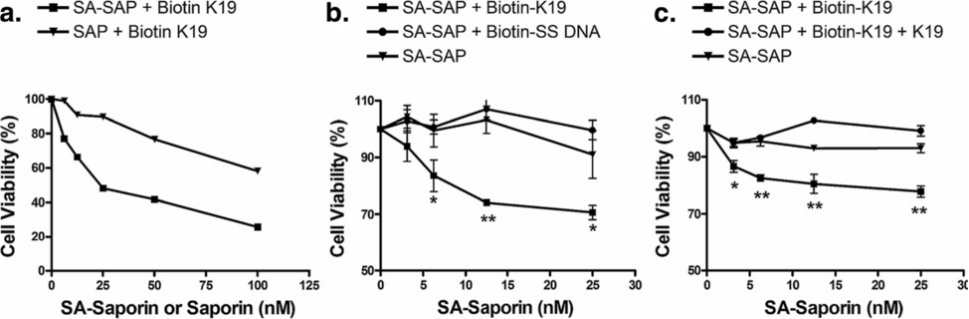
**Using Siglec-5 aptamer K19 to enhance the saporin toxicity for inhibiting NB4 cell proliferation in vitro.** Various concentrations of biotinylated-aptamer K19 (Biotin-K19) or biotinylated single-stranded negative control DNA (Biotin-SS DNA) and streptavidin-saporin (SA-SAP) or free saporin (SAP) were mixed, and were then incubated with NB4 cells **(aandb)**. The non-biotinylated aptamer K19 (K19) was used to block the binding of biotin-K19 to NB4 cells **(c)**. The viable cells were then measured in 72 hours. The experiments were performed in triplicates, and the data is shown as fractions of the control groups. The *P* values are given as “*” or “**” representing the *P* values of < 0.05 or < 0.01, respectively.

Because Siglec-5 is overexpressed in a subset of AML cells, we selected an AML case with relatively high levels of Siglec-5 expression, and spiked small numbers of the AML cells into a normal human bone marrow specimen. Then, based on the differential expression levels of Siglec-5 on normal CD34(+) cells and CD34 (+) leukemic cells, we used aptamer K19 to aid in the detection of AML cells mixed into a normal bone marrow specimen (Figure 8). Additionally, to demonstrate Siglec-5 can be a potential biomarker for targeted therapy, we tested biotinylated Siglec-5 aptamer K19 and saporin-cross-linked to streptavidin (SA-SAP) for inhibiting NB4 cell proliferation in vitro. Compared with unlabeled saporin (SAP) (Figure 9a) or the biotinylated single stranded DNA control (Figure 9b), the Siglec-5 aptamer K19 can enhance the toxicity of SA-SAP to NB4 cells with an estimated IC50 of 25 to 50 nM. The enhanced toxic effect of biotinylated K19 aptamer can be blocked by non-labelled aptamer K19, indicating that the enhanced cell toxicity is mediated through the specific binding to surface Siglec-5 proteins (Figure 9c).

## Discussion

The molecular characteristics of leukemic cells, especially at the proteomic level, are critical for understanding leukemia pathogenesis and designing targeted therapy. In the last several decades, proteomic analysis has been performed to advance the discovery of diseased cell-specific protein biomarkers, but so far only a few AML biomarkers have been introduced into clinical practice for AML detection and therapy. Currently, we still lack effective biomarkers for AML diagnosis and targeted therapy. Thus, our intent in this study is to develop new molecular probes that target surface protein biomarkers on AML cells.

Membrane proteins function as adhesion-anchors, receptors, transporters and enzymes that play roles in various physiological processes, and their dysregulation may contribute to the pathogenesis of many disease processes, including AML. It is estimated that 20–35% of the mammalian genome encodes membrane proteins [30]. However, membrane proteins remain mostly under-represented in the proteomic analysis due to technical challenges. Proteomic studies, using mass spectrometry-based technology, aim at identifying individual proteins so that an assay, most often antibody-based, can be developed for a specific protein. By contrast, the Cell-SELEX approach produces a group of cell-specific aptamers that can be verified in clinical specimens without purified proteins or even knowledge of their protein targets. The selected aptamers can easily be labelled for flow cytometry or image analysis of cells in clinical specimens. If one of the aptamer probes detects a surface marker of interest, the specific aptamer probe can be used to enrich and purify the target protein, such as aptamer K19 and its target Siglec-5. It should be noted that our studies do not seek to compare the capability of aptamers with antibodies. Numerous high quality monoclonal antibodies have been produced for isolated proteins. However, it is difficult to make antibodies when we do not know the target proteins on tumor cells.

We used HL-60 and NB4 human leukemic cell lines for our experiments, and the two cell lines are closely related. While both can differentiate under chemical induction, only the NB4 cells originated from AML M3, and carry the t(15;17) chromosome translocation [31-33]. By using these two leukemic cell lines, we can address three questions: A). Is it possible to select single stranded DNA aptamers that are capable of detecting differences in surface protein expression between two closely related leukemic cell lines (HL60 and NB4)? B). Can these selected aptamers be further used on clinical specimens for phenotyping AML and identifying new biomarkers? C). Can the newly identified biomarker be used to aid the detection of AML cells in human bone marrow specimens?

As a result, we used NB4 leukemic cells to select and characterize three new DNA aptamers (JH6, JH19, and K19), which have more binding sites on NB4 cells than on HL60 cells. This is in contrast to the aptamer KH1C12 previously selected from HL60 cells, which selectively recognized HL60 cells [19]. Gene expression profiling studies showed that NB4 and HL60 cell lines had the most closely related profiles of mRNA expression [33]. Thus, our results with aptamers selected against NB4 and those previously selected against HL60 cells indicate that it is possible to select aptamers capable of detecting differences in surface protein expression between two closely related leukemic cell lines.

The fluorescence intensity levels of bound aptamers on leukemic cells, compared with those on CD34(+) progenitors in normal bone marrow specimens, vary significantly among different AML cases (Figure 3). The findings are likely reflective of the heterogeneity of disease in the AML group. While the overall fluorescence levels of bound aptamers between the CD34(+) normal progenitors and AML non-M3 groups are not statistically significant, subsets of AML non-M3 cases may overexpress one or more surface biomarkers that can be recognized by aptamers. The heterogeneity of AML requires great effort and resources in order to develop useful biomarkers for its detection and treatment, but we can also use the heterogeneity of biomarker expression for diagnosis or targeted therapy. If an AML case overexpresses one or more surface biomarkers that can be recognized by aptamers (e.g. Siglec-5 in Figure 8), the aptamer probes may become useful tools for detecting the minimal residual disease of AML after chemotherapy.

Despite their selection from NB4 cells derived from a case of AML M3 and their ability to recognize maturing granulocytes and monocytes well (Figure 2), all three aptamers show relatively lower levels of binding to AML M3 (Figure 3). Significant down-regulation of normal myeloid markers may occur on AML M3 cells in clinical specimens, and one well-known example is CD15. Like CD15, the aberrantly down-regulated expression of aptamer target proteins, including Siglec-5 in AML M3, makes it possible for us to use them as biomarkers to differentiate between AML non-M3 and AML M3 cases in clinical practice. Conversely, if AML cells in a clinical specimen show high levels of reactivity to the three aptamers, the case is unlikely to represent AML M3.

The human, CD33-related, sialic acid binding, immunoglobulin-like lectins (CD33rSiglecs) comprise a family of receptors (including Siglec-5) that are differentially expressed on leukocytes. The aptamer K19, which recognizes Siglec-5, can recognize granulocytes and monocytes, with no significant binding to bone marrow lymphocytes (Figure 2). In addition, with aptamer K19 we demonstrated relatively low levels of Siglec-5 on CD34(+) progenitor cells in normal bone marrow (Figure 2), and up-regulated Siglec-5 during the granulocytic maturation (Figure 7). These results are consistent with previously reported results that Siglec-5 expression was up-regulated later than CD33 during *in vitro* myeloid differentiation of CD34(+) cells purified from cord blood [26].

CD33 has been used as one of the common markers of AML. The antibody against CD33, gemtuzumab (Mylotarg™), has been tested for the treatment of AML, and was reported to be effective at inducing remissions in about 25-30% of relapsed AML patients despite its reversible toxicity on normal myeloid cells [34]. Due to the restricted expression of CD33 on different leukocyte cell types, other members of the CD33rSiglecs family, including Siglec-5, have also been explored as targets for cell-directed therapies of AML [35]. It was shown that anti-Siglec-5 antibody linked with saporin toxin induced cell killing in U937 human leukemic cells [36]. Our studies show that the Siglec-5 aptamer K19 can compete with anti-Siglec-5 antibody for binding to NB4 cells (Figure 6), and through Siglec-5 proteins it can also mediate uptake of Saporin to inhibit NB4 cell proliferation in vitro. The published results of immunotoxin studies were performed with antibodies directly conjugated to toxin ([37,38]), and it should be noted that Saporin is linked to streptavidin in our studies rather than directly conjugated to aptamer K19. Therefore, the efficiency of aptamer-mediated up-take of saporin might be low. In these studies, our intent is to demonstrate the potential of Siglect-5 and its aptamers, and it is necessary to perform further optimization of aptamers and aptamer-toxin conjugates in order to determine whether Siglec-5 and its aptamer can truly be used as a biomarker for detection and targeted therapy of AML.

In summary, in this reported study, we have demonstrated a pipeline approach for biomarker discovery. We first employed the Cell-SELEX technique to select DNA aptamers that can be used as molecular probes to phenotype normal hematopoietic cells and AML cells. We then used one of the aptamers to enrich and identify its target protein on the surface of leukemic cells. Finally, we demonstrated that the identified biomarker (Siglec-5) can aid in the detection of AML cells at low concentrations, and can potentially mediate targeted therapy of AML cells. This strategy developed with leukemic cells should be applicable to other types of cancer to facilitate biomarker discovery and targeted cancer therapy.

## Competing interests

The authors declare no competing financial interests.

## Authors’ contributions

MY and GJ, performed research and analyzed the data and wrote the paper; WL, KQ and MZ, performed research work; SA and CC, performed leukemia data analysis and wrote the paper; YL, designed research, analyzed the data, and wrote the paper. All authors read and approved the final manuscript.

## Supplementary Material

Additional file 1: Figure S1 Flow cytometry assay for monitoring enrichment of the specific aptamer pool against NB4 leukemia cells. After 10 rounds of selection processes, the phycoerythrin (PE) labeled aptamer pool showed significant increases in fluorescence intensity on target NB4 cells, but it produced minimal change in fluorescence intensity on HL60 cells. These results indicate that the aptamers recognizing target NB4 cells were enriched preferentially. Click here for file
