# Supplementary material for: Developing aptamer probes for acute myelogenous leukemia detection and surface protein biomarker discovery
Source: J Hematol Oncol. 2014 Jan 9;7:5. doi: 10.1186/1756-8722-7-5 (PMC3895837; doi:10.1186/1756-8722-7-5)
Supplement: Additional file 1: Figure S1 — Flow cytometry assay for monitoring enrichment of the specific aptamer pool against NB4 leukemia cells. After 10 rounds of selection processes, the phycoerythrin (PE) labeled aptamer pool showed significant increases in fluorescence intensity on target NB4 cells, but it produced minimal change in fluorescence intensity on HL60 cells. These results indicate that the aptamers recognizing target NB4 cells were enriched preferentially. [file 1756-8722-7-5-S1.pdf]

Supplemental data:

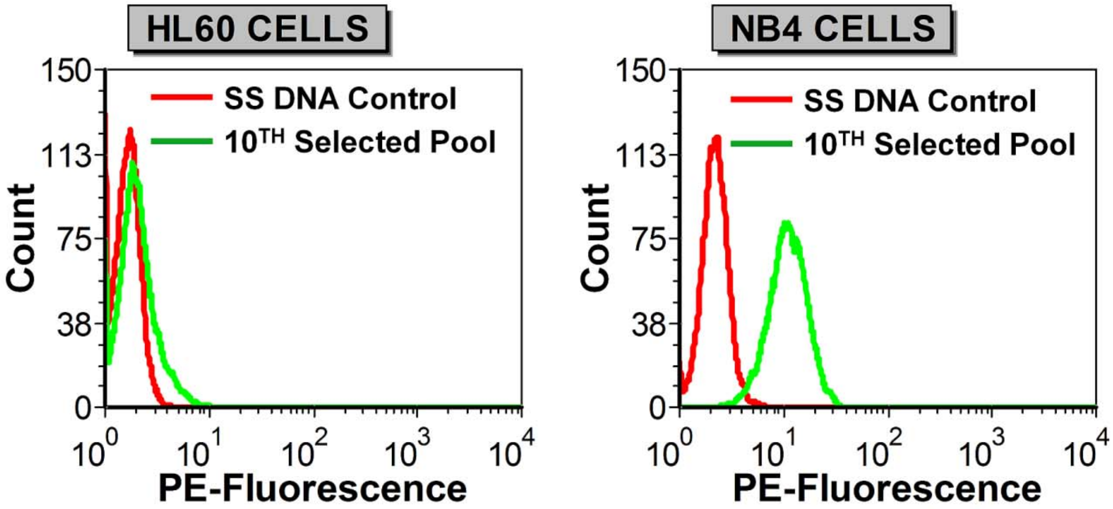

Figure S1. **Flow cytometry assay for monitoring enrichment of the specific aptamer pool against NB4 leukemia cells.** After 10 rounds of selection processes, the phycoerythrin (PE) labeled aptamer pool showed significant increases in fluorescence intensity on target NB4 cells, but it produced minimal change in fluorescence intensity on HL60 cells. These results indicate that the aptamers recognizing target NB4 cells were enriched preferentially.
